# Global Fibrosis Burden and a Transcriptional Biomarker-Based Strategy for Early Detection in Resource-Limited Settings

**DOI:** 10.3390/biom15091273

**Published:** 2025-09-03

**Authors:** Qinqin Deng, Longjiang Wu, Chenlu Zhang, Mei Dang

**Affiliations:** 1 College of Biological Sciences and Engineering, Shaanxi University of Technology, Hanzhong 723000, China; dengqinqin@snut.edu.cn (Q.D.); longjiang_wu1999@163.com (L.W.); 2 Shaanxi Provincial Key Laboratory of Resource Biology, Shaanxi University of Technology, Hanzhong 723000, China

**Keywords:** fibrosis, global burden of disease (GBD), vestigial-like family member 3 (VGLL3), early diagnosis, health disparities, protein biomarkers, intrinsically disordered proteins, resource-limited settings

## Abstract

Fibrotic diseases contribute to nearly half of all deaths in industrialized countries, yet effective early detection strategies remain lacking, particularly in low-resource settings. This study aimed to quantify the global burden of fibrosis-related diseases using updated global burden of disease (GBD) 2021 data across 204 countries and territories and establish a cost-effective diagnostic approach targeting vestigial-like family member 3 (VGLL3), a fibrosis-associated transcriptional co-regulator. Our analysis revealed that from 1990 to 2021, fibrosis-related disability-adjusted life years (DALYs) and mortality increased by 16.71% and 4.83%, respectively, with neoplasms and chronic obstructive pulmonary disease (COPD) being the main contributors. We also found a growing burden disproportionately concentrated in low socio-demographic index (SDI) regions. To address the diagnostic gap, we developed a novel immunoassay targeting VGLL3, an intrinsically disordered transcriptional co-regulator implicated in early fibrotic remodeling. The assay demonstrated a detection range of 27.01–2512.36 nM and a limit of detection of 12.55 nM. Immunohistochemical validation in a mouse myocardial infarction model confirmed the antibody’s specificity in fibrotic tissues. This work highlights widening global health disparities in fibrosis burden and introduces a cost-effective, scalable diagnostic strategy for early fibrosis detection, particularly suitable for resource-limited settings.

## 1. Introduction

Fibrosis is a progressive pathological process characterized by excessive deposition of extracellular matrix (ECM) [1], which causes diseases such as metabolic dysfunction-associated steatohepatitis, inflammatory bowel disease, chronic kidney disease, idiopathic pulmonary fibrosis, and systemic sclerosis [2]. It is estimated that fibrotic complications contribute to nearly 45% of all deaths in industrialized countries [3]. With population aging, environmental exposures, and chronic disease prevalence continuing to rise, the burden of fibrosis is expected to increase further.

Understanding global fibrosis epidemiology is essential for guiding prevention and intervention strategies. The Global Burden of Disease (GBD) study provides a unique platform for tracking disease burden over time and across regions [4]. However, fibrosis-specific analysis has often been limited by its classification under broader non-communicable disease categories. With the release of GBD 2021, there is now an opportunity to reassess temporal trends and inequality patterns in fibrosis burden using more recent and detailed data. Our analysis shows that the global burden of disability-adjusted life years (DALYs) associated with fibrosis is increasing, particularly in regions with a lower socio-demographic index (SDI). These regions not only face inadequate medical facilities and high-risk environmental exposures but also lack effective screening methods. This prevents many fibrosis patients from timely diagnosis and treatment, further exacerbating regional health disparities.

Despite its clinical importance, the timely detection of fibrosis remains a significant challenge. Conventional methods of detecting fibrosis include cardiac magnetic resonance imaging, echocardiography, and histopathology [5]. These methods are usually organ-specific, expensive, and difficult to apply early in resource-limited areas, often leading to delayed intervention and missed reversible windows. Therefore, a molecular marker that can reflect the early molecular events of fibrosis, has cross-organ applicability, and is scalable is urgently needed. VGLL3 is a transcriptional coregulator rich in intrinsically disordered regions (IDRs) that is consistently upregulated in multi-organ fibrosis and mediates early activation of fibroblasts [6]. Due to its structural features and broad expression pattern, VGLL3 has become a candidate biomarker of interest in this study.

Studies have shown that VGLL3 can respond to changes in matrix stiffness, bind to TEAD, directly mediate fibroblast collagen production, play a key role in liver fibrosis, and its mRNA expression is significantly upregulated in fibrotic liver tissue of patients with non-alcoholic fatty liver disease (GSE126848) [6]. In cardiac fibrosis, VGLL3 knockout significantly reduced the degree of fibrosis after myocardial infarction in mice and improved cardiac function [6]. In keloids, VGLL3 promotes fibroblast proliferation by activating the Wnt pathway [7]. In addition, VGLL3 is upregulated in normal skin fibroblasts under TGF-β stimulation and promotes skin fibrosis [8]. These studies demonstrate the potential of VGLL3 as a biomarker at the fibrotic tissue level. Unlike traditional ECM biomarkers that reflect late-stage fibrotic remodeling, VGLL3 plays a role in the early stages of fibroblast activation and has the potential to serve as a tissue-level biomarker for early diagnosis and mechanism intervention.

To bridge the gap between global fibrosis surveillance and practical diagnostics, we employed a two-pronged approach. First, we performed a comprehensive analysis of global fibrosis burden using GBD 2021 data, with a focus on temporal trends, major disease drivers, and inequality patterns. Second, we developed a VGLL3-targeted diagnostic platform based on avian antibodies, a cost-effective and scalable alternative to mammalian IgG with superior stability and specificity [9,10]. A VGLL3-specific avian antibody was generated, and an indirect competitive enzyme-linked immunosorbent assay (ic-ELISA) was established with high sensitivity, validated both in vitro and in a mouse myocardial fibrosis model. By integrating epidemiological insights with diagnostic innovation, this study provides a foundation for targeted fibrosis surveillance and early detection strategies, particularly for resource-limited settings facing the heaviest burden.

## 2. Materials and Methods

### 2.1. Global Burden Analysis Based on GBD 2021

To evaluate the global burden and disparities associated with fibrosis-related diseases, we extracted data from the GBD 2021 database available through the Institute for Health Metrics and Evaluation website (http://ghdx.healthdata.org/ accessed on 8 May 2025). This dataset includes standardized epidemiological estimates for 204 countries and territories, integrating data from multiple sources such as vital registration systems, health surveys and administrative records. DALYs were used as the primary metric to quantify the burden attributable to major fibrotic drivers, particularly neoplasms and chronic obstructive pulmonary disease (COPD). Analysis was stratified by SDI to facilitate comparisons across low-, middle-, and high-income settings. All statistical analyses were performed using R software (version 4.4.2), with *p*-values < 0.05 considered statistically significant.

#### 2.1.1. Inequality Analysis

To quantify socioeconomic disparities in fibrosis-related disease burden, we conducted a two-part health inequality assessment based on SDI. Countries were ranked by SDI scores and assigned percentile ranks. The slope index of inequality was calculated by regressing DALYs rates against SDI ranks, using the population-weighted midpoint of the cumulative SDI distribution. Robust linear regression was used to control for outliers and heterogeneity. The concentration index of inequality was calculated by comparing the cumulative proportion of DALYs with the cumulative population distribution ordered by SDI, using numerical integration of the Lorenz curve area.

#### 2.1.2. Frontier Analysis

To evaluate whether countries’ fibrosis-related DALYs rates aligned with their level of development, we performed a frontier efficiency analysis. A non-parametric locally weighted regression was used to estimate the empirical frontier relationship between age-standardized rate (ASR) of DALYs and SDI scores from 1990 to 2021. This frontier represents the theoretical minimum ASR of DALYs achievable at a given SDI level. The vertical gap between each country’s actual DALYs rate and the frontier was calculated as a proxy for performance deviation, with countries close to the frontier considered to have maximized their potential in reducing fibrosis burden relative to their socioeconomic development.

### 2.2. VGLL3 Antigen Design, Expression, and Purification

VGLL3 sequence data were downloaded from UniProt database accession number P85442. To construct the VGLL3 antigen for immunization, the full-length VGLL3 coding sequence was cloned into the pET-28a (+) vector using NcoI and XhoI restriction sites. An N-terminal SUMO tag and TEV protease cleavage site were included to enhance protein solubility and facilitate purification. In total, five truncated VGLL3 variants (residues 1–168, 1–194, 1–237, 1–251, and 1–297) were designed based on domain prediction and cloned similarly using sequence-specific primers (Table S1). Recombinant plasmids were transformed into *E. coli HST08* cells and verified by sequencing (Tsingke Biotech, Xi’an, China).

Among these constructs, the VGLL3 (1–237) fragment demonstrated good expression in *E. coli* while retaining key structural domains. This construct was transformed into *E. coli BL21 (DE3)* for protein production. Cultures were grown in LB medium with 50 µg/mL kanamycin at 37 °C until OD600 reached 0.6–0.8, followed by induction with 0.5 mM IPTG for 8 h. Cells were harvested, washed in PBS, and lysed in 8 M urea buffer (pH 7.6) supplemented with protease inhibitors. The lysate was sonicated and centrifuged at 12,000 rpm for 1 h. The supernatant was purified by Ni-NTA affinity chromatography (Thermo Fisher Scientific, Waltham, MA, USA), eluted with 25% imidazole in 8 M urea, and dialyzed overnight in sterile water at 4 °C. Protein integrity was confirmed by SDS-PAGE, and aliquots were stored at −80 °C for downstream applications.

### 2.3. Chicken Immunization, Avian Antibody Preparation, and Antibody Validation

VGLL3-specific polyclonal avian antibody was generated by immunizing 20-week-old laying hens with the purified VGLL3 (1–237) antigen. The initial dose of 250 µg was emulsified in Freund’s complete adjuvant (Sigma-Aldrich, St. Louis, MO, USA) and administered via intramuscular injection. Three booster immunizations (200 µg each) were given at two-week intervals using Freund’s incomplete adjuvant. Eggs were collected and stored at 4 °C.

Laying hens were kept in a chicken house at (20 ± 2) °C and relative humidity (60 ± 5%), with 3 hens per cage. They were given complete feed and sufficient drinking water, and the light was on for 16 h per day. During immunization, they were gently restrained. If the hens showed pain-related behaviors such as depression and reduced food intake, an appropriate amount of butorphanol was injected intramuscularly in time for analgesia.

Avian antibodies were extracted from egg yolks using polyethylene glycol precipitation [11]. Purity was confirmed by SDS-PAGE. To further validate the specificity of the avian antibodies, Western blot analysis was performed. Different loading volumes (5–20 µL) of VGLL3 (1–237) protein were separated by 12% SDS-PAGE and transferred to PVDF membranes (Merck, Kenilworth, NJ, USA). The membrane was blocked with TBST buffer containing 5% non-fat milk overnight at 4 °C and then incubated with the anti-VGLL3 avian antibody (1:1000 in TBST) at 37 °C for 1 h. After washing with TBST 5 times for 10 min each time, the membranes were incubated with an HRP-conjugated rabbit anti-chicken secondary antibody (SinoBiological, Beijing, China) for 1 h at 37 °C. Signals were detected using the BevoECL Moon chemiluminescent kit (Beyotime Biotechnology, Shanghai, China) and visualized using a chemiluminescent imaging system (Bio-Rad Laboratories, Hercules, CA, USA).

### 2.4. Antibody Binding Analysis by ic-ELISA

Optimal conditions for ic-ELISA were established using checkerboard titration [12]. Transparent microtiter plates (Costar Inc., Cambridge, MA, USA) were coated overnight with the truncated VGLL3 protein (10 μg/mL, 100 μL/well) in PBS. After three washes with PBS-T, wells were blocked with 200 μL of 5% skim milk powder in PBS at 37 °C for 2 h. Serial dilutions of truncated VGLL3 (0.25–20 µg/mL) were mixed 1:1 with the anti-VGLL3 avian antibody (1:1600), and 100 μL of the mixture was added per well. Following a 1 h incubation at 4 °C and subsequent washing, rabbit anti-chicken HRP-conjugated secondary antibody (1:5000 in PBS with 2% skim milk powder) was applied (100 μL/well) and incubated at 37 °C for 1 h. After additional washes, 3,3′,5,5′-tetramethylbenzidine substrate solution (100 μL/well) was added, and plates were incubated at 37 °C for 10 min. The reaction was stopped with 50 μL of 2 M H_2_SO_4_, and absorbance was measured at 450 nm using an ELISA plate reader (BioTek Instruments Inc., Winooski, VT, USA). Competitive inhibition curves were generated and fitted to a logistic equation using Origin 2021 (OriginLab, Northampton, MA, USA).

### 2.5. Myocardial Infarction Model and Immunohistochemistry

To validate the antibody’s performance in tissue, a mouse model of myocardial infarction was established in 8-week-old C57BL/6 mice (Shanghai Fuyan Biotechnology Co., Shanghai, China). Mice were anesthetized and ventilated via oral intubation. A left thoracotomy was performed to expose the heart, and the left anterior descending coronary artery was ligated with 4–0 silk sutures to induce ischemia, confirmed by myocardial blanching. After closure and recovery, mice were monitored for four weeks. All animal procedures were approved by Shanghai Fuyan Biotechnology Corporation (Shanghai, China) and conformed to national animal welfare regulations.

At endpoint, mice were euthanized by cervical dislocation, and heart tissues were harvested, fixed in 4% paraformaldehyde, and embedded in paraffin. Sections (5 μm) were deparaffinized, rehydrated, and subjected to antigen retrieval in Tris-EDTA buffer (pH 9.0). Endogenous peroxidase activity was quenched, and sections were blocked with 5% BSA. Primary anti-VGLL3 avian antibody (1:1000) was applied overnight at 4 °C, followed by incubation with a biotin-labeled anti-chicken secondary antibody (1:2000). Signal was developed using DAB substrate (Thermo Fisher Scientific, Waltham, MA, USA) and counterstained with hematoxylin. Slides were dehydrated and mounted for microscopic analysis (Nikon Eclipse Ci-L, Tokyo, Japan).

### 2.6. VGLL3 Detection in Human Plasma Samples

Plasma samples were obtained from two female patients with clinically confirmed liver cirrhosis admitted to Hanzhong Central Hospital. Blood was collected in EDTA-anticoagulant tubes and centrifuged at 1000 rpm for 10 min to separate plasma. 25–35 μL of each plasma sample was separated by 12% SDS-PAGE electrophoresis and transferred to a PVDF membrane. Blocking, antibody incubation, and color development conditions were the same as those for Western blot in Section 2.3. Detection was performed using the VGLL3-specific avian antibody (1:1000) and an HRP-conjugated rabbit anti-chicken secondary antibody (1:2000). Development was performed using the BevoECL Moon chemiluminescence assay (Beyotime Biotechnology, Shanghai, China).

## 3. Results

### 3.1. Temporal Trends in the Proportion of Global Fibrosis-Related DALYs

**Figure 1:**
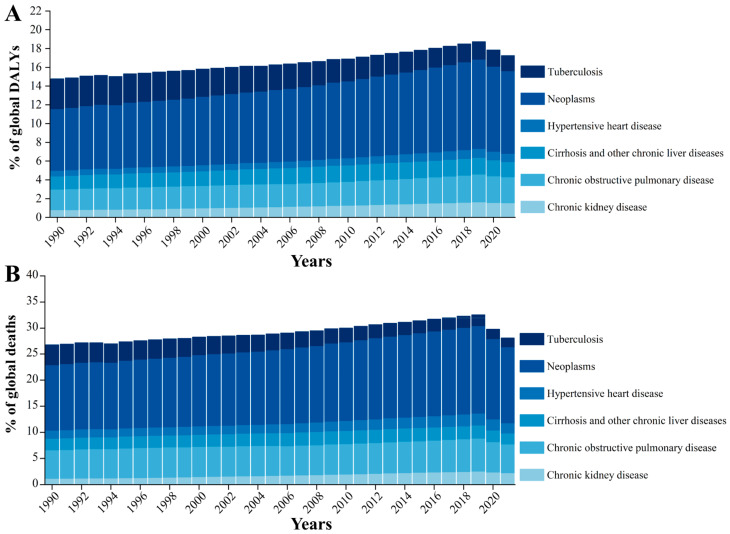
Temporal trends in the proportion of global disability-adjusted life years (DALYs) (**A**) and deaths (**B**) attributable to fibrosis-related diseases (1990–2021). Stacked bar chart illustrating the annual percentage of DALYs attributable to major fibrosis-associated conditions from 1990 to 2021. The analysis includes neoplasms, chronic obstructive pulmonary disease (COPD), chronic kidney disease, cirrhosis and other chronic liver diseases, hypertensive heart disease, and tuberculosis.

Between 1990 and 2021, the global burden of fibrosis-related diseases exhibited a steadily increasing trend in both DALYs and mortality rates. The proportion of DALYs attributable to fibrosis-related conditions rose from 14.78% to 17.25%, reflecting a relative increase of approximately 16.71%. Similarly, the mortality rate increased from 26.78% to 28.08%, representing a 4.83% rise. Among various pathogenic factors, neoplasms consistently accounted for the largest share, with their contribution to DALYs rising from 6.57% in 1990 to 8.80% in 2021, and to mortality from 12.55% to 14.57%. COPD remained the second leading cause throughout the period, with its DALY share increasing from 2.20% to 2.77%, and a slight rise in mortality from 5.41% to 5.48% (Figure 1).

Although chronic kidney disease represented a smaller proportion of the total burden, it exhibited the most rapid relative growth, with its DALYs and mortality rates increasing by 92.47% and 87.75%, respectively. In contrast, tuberculosis was the only major cause of disease burden to experience a significant decline, with its mortality rate falling by 214.55% compared to 1990. For cirrhosis and other chronic liver diseases, the proportion of DALYs increased modestly from 1.40% to 1.61%, while mortality declined from 2.22% to 2.10%. Hypertensive heart disease remained a minor contributor to fibrosis-associated burden in 2021, accounting for 0.88% of DALYs and 1.96% of deaths globally (Figure 1).

### 3.2. Regional Disparities and Temporal Trends in Fibrosis-Related Burden Attributable to Neoplasms and COPD

**Figure 2:**
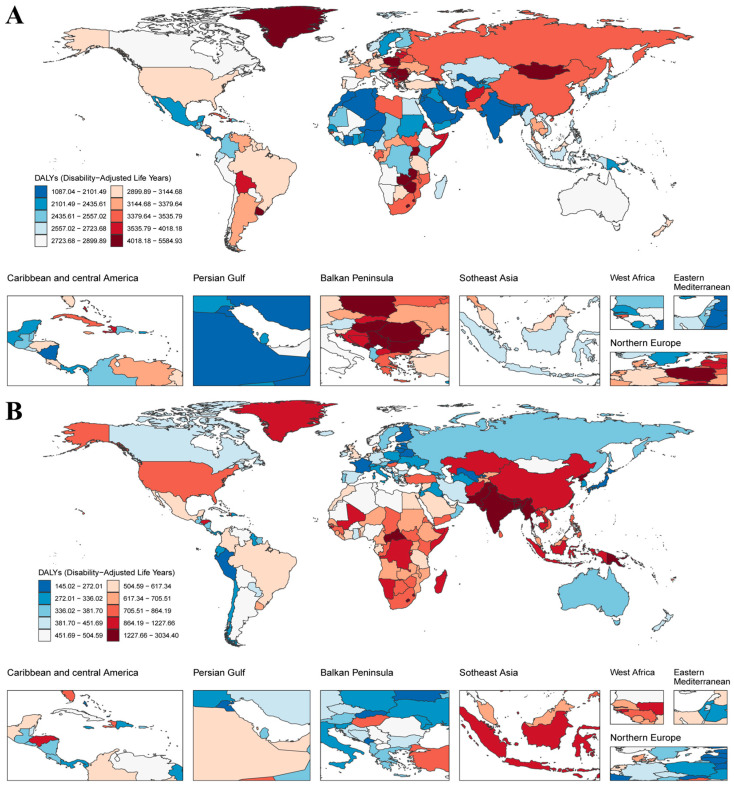
Geographic distribution, regional disparities, and socio-demographic index (SDI)-based projections of fibrosis-related DALYs due to neoplasms and COPD (1990–2021). Global maps of age-standardized rate (ASR) per 100,000 population due to neoplasms (**A**) and COPD (**B**) in 2021. Insets highlight regional hotspots, including the Caribbean, Persian Gulf, Balkan Peninsula, Southeast Asia, West Africa, and the Eastern Mediterranean.

Marked regional disparities were observed in the fibrosis-related burden of neoplasms and COPD, the two leading contributors to global fibrosis-associated disability. In 2021, the ASR of neoplasm DALYs was highest in Europe, especially in Central Europe, with 3945.02 cases per 100,000 population (95% UI: 3668.14, 4199.24), followed by southern sub-Saharan Africa and East Asia (Figure 2A and Table S2). The highest burden of COPD was concentrated in Oceania (2351.49 cases per 100,000 population, 95% UI: 1931.26, 2854.06) and South Asia (2049.22 cases per 100,000 population, 95% UI: 1862.71, 2268.73) (Figure 2B and Table S3). Notably, there were regional differences in the findings: the Balkan Peninsula and Northern Europe countries had a high neoplasms-related burden but a low COPD-related burden, whereas East and South Asian countries showed the opposite trend (Figure 2).

Between 1990 and 2021, the global ASR of neoplasm-related DALYs declined by 25.59%. High-middle SDI countries reported the highest DALYs rates in 2021 (3388.27 per 100,000) despite a reduction in age-standardized death rate to 134.01 per 100,000 (Table 1). These regions also experienced the most pronounced improvements in DALYs rates. In contrast, low-middle and low SDI regions demonstrated slower progress. While the 2021 ASDR in these groups were relatively low (85.03 and 90.64 per 100,000, respectively), their ASR of DALYs declined only marginally (by 1.33% and 13.17%, respectively) over the 31-year period. This persistent gap underscores disparities between disease burden and healthcare capacity in lower-income settings.

For COPD, global disease burden also declined from 1990 to 2021. Total COPD-related deaths dropped by 37.12% (from 71.92 per 100,000 population to 45.22 deaths), and the ASR of DALYs decreased by 36.98% (from 1492.64 to 940.66 per 100,000) (Table 1). The most substantial reductions occurred in high-middle SDI countries, where the ASDR fell by 54.85% and the ASR of DALYs by 54.27%. Middle SDI regions followed with comparable reductions of 53.63% in ASDR and 53.85% in ASR of DALYs. Despite this progress, the COPD burden remains disproportionately high in low-SDI regions, where the 2021 ASR of DALYs (1457.94 per 100,000) was more than three times that in high-SDI regions (471.22 per 100,000) (Table 1). Regionally, East Asia exhibited the steepest reduction in COPD-related ASR of DALYs (67.62%) yet still exceeded the global average (Table S3).

### 3.3. Inequality Analysis Reveals Persistent Socioeconomic and Regional Disparities in Fibrosis-Related Burden

**Figure 3:**
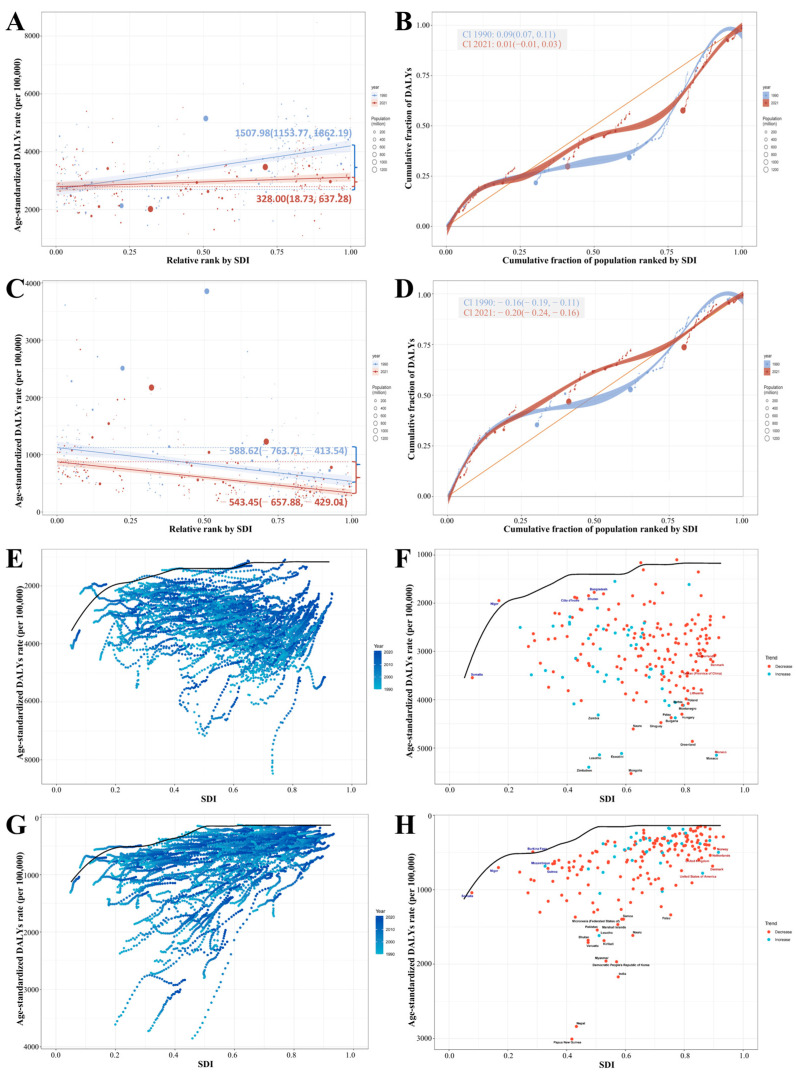
Global inequality and frontier analysis of DALY burden of fibrosis-related neoplasms and COPD (1990–2021). The slope index of inequality plots showing the relationship between ASR of DALYs and relative SDI rank across 204 countries and territories for neoplasms (**A**) and COPD (**C**) in 1990 and 2021. Dots represent countries, and line slopes reflect absolute inequality. The concentration index of inequality plots comparing cumulative population share (ranked by SDI) with cumulative DALYs share for neoplasms (**B**) and COPD (**D**) from 1990 to 2021. SDI-DALYs time trajectories from 1990 to 2021 (light to dark blue), depicting each country’s DALYs rate evolution for neoplasms (**E**) and COPD (**G**) relative to their SDI progression. Scatter plots show the deviation from the model minimum DALYs rate (black curve) for neoplasms (**F**) and COPD (**H**) at a given SDI for each country. Each dot corresponds to a specific country or region, and the top 15 countries and regions with the largest deviations from that border are also highlighted in black font. Blue labels represent low-SDI countries with the smallest deviations from the border, and red fonts represent high-SDI countries with the largest deviations from the border. The direction of change in ASR of DALYs from 1990 to 2021 is indicated by the color of the dot, with red dots indicating a decline and cyan dots indicating an increase.

Inequality analysis revealed persistent and distinct disparities in fibrosis-related disease burden across socioeconomic levels, particularly for neoplasms and COPD. For neoplasms, the slope index of inequality decreased markedly from 1507.98 (95% UI: 1153.77–1862.19) in 1990 to 328.00 (18.73–637.28) in 2021 (Figure 3A), indicating a substantial reduction in absolute disparities between high- and low-SDI countries. Similarly, the concentration index of inequality dropped from 0.09 to 0.01 over the same period (Figure 3B), suggesting a shift from a burden skewed toward high-SDI countries to a more balanced global distribution. In contrast, the slope index of inequality for COPD narrowed slightly from −588.62 (−763.71 to −413.54) in 1990 to −543.45 (−657.88 to −429.01) in 2021 (Figure 3C), the concentration index of inequality became more negative, declining from −0.16 to −0.20 (Figure 3D). This reflects a worsening concentration of DALYs in low-SDI regions, indicating widening health inequality despite modest improvements in absolute burden. These findings highlight a troubling divergence: while neoplasm-related disparities are declining globally, COPD-related burden is increasingly concentrated in disadvantaged regions.

To further elucidate the relationship between socioeconomic development and fibrosis-related disease burden, we conducted frontier analysis. Overall, ASR of DALYs for both neoplasms and COPD declined across most high-, high-middle-, and middle-SDI countries between 1990 and 2021 (Figure 3E,G). However, trajectories varied markedly across nations. Notably, several low-SDI regions including Somalia, Niger, Côte d’Ivoire, Bhutan, Bangladesh, Burkina Faso, Mozambique, and Guinea have demonstrated substantial success in reducing fibrosis-related burden, approaching the global best-performing frontier. Conversely, several high-SDI regions including Netherlands, Denmark, Taiwan (province of China), Lithuania, Norway, the United Kingdom, and the United States still exhibit considerable room for improvement (Figure 3F,H). Together, these results reinforce the need for context-specific strategies that address not only absolute burden but also inequality and inefficiency in fibrosis-related disease control.

### 3.4. Development and Validation of a VGLL3-Targeted Avian Antibody for Fibrosis Detection

**Figure 4:**
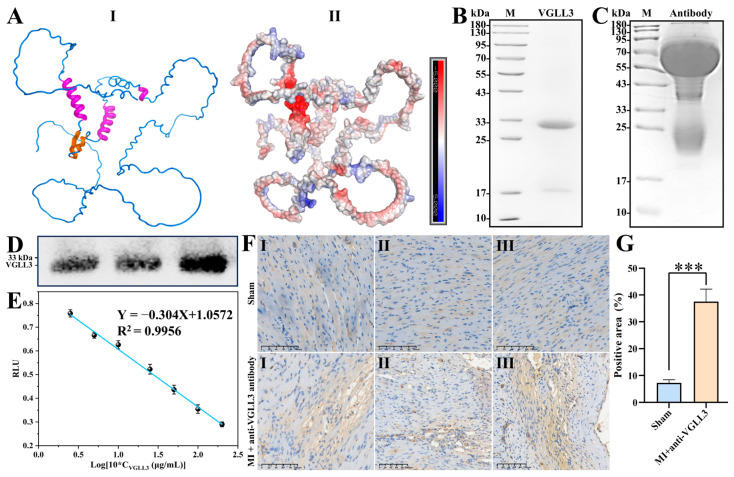
Structural modeling, antibody production, and immunodetection of vestigial-like family member 3 (VGLL3) in myocardial fibrotic tissue. (**A**) Structural prediction of mouse VGLL3 using AlphaFold2 online server with default parameter settings. (I) Cartoon representation highlighting α-helices (magenta), β-strands (orange), and other regions (marine). (II) Electrostatic surface potential map, with red indicating negatively charged regions and blue indicating positively charged regions. (**B**) SDS-PAGE analysis of the purified VGLL3 (residues 1–237). (**C**) SDS-PAGE analysis of the purified anti-VGLL3 avian antibody. Lane M: protein marker. (**D**) Western blot analysis of anti-VGLL3 avian antibody specificity, with protein loading amounts of 10, 15, and 25 μL. (**E**) Standard curve for indirect competitive enzyme-linked immunosorbent assay (ic-ELISA) detection. Relative light units (RLU) are expressed as the ratio of sample signal (B) to control signal (B_0_). (**F**) Representative images of cardiac tissue sections from the sham group (top) and the anti-VGLL3 avian antibody-stained myocardial infarction group (bottom). Scale bar = 100 μm. (**G**) Quantification of VGLL3-positive staining area in sham and myocardial infarction groups. Data represent mean ± SEM. *** *p* < 0.001 by unpaired *t*-test (n = 3 per group). Original images can be found in Supplementary Materials.

Considering the crucial role of VGLL3 in fibrosis, we posited that it could serve as a potential target for diagnosis and therapy. Initial attempts to express full-length VGLL3 in vitro resulted in extremely low expression levels, even with solubility-enhancing strategies such as SUMO tag fusion, and insufficient for subsequent experiments (Figure S1A,B). Structural prediction using AlphaFold2 revealed that the VGLL3 protein contains two α-helices, two β-sheets, and numerous IDRs, some of which exhibit a propensity for nascent helices (Figure 4A). Based on these structural predictions, we designed five truncated VGLL3 constructs (corresponding to residues 1–168, 1–194, 1–237, 1–251, and 1–297) to retain key structural elements (Figure S1D). Small-scale expression and Western blot analysis with an anti-(His)_6_-tag antibody demonstrated successful expression of all five truncated constructs (Figure S1A,B). Among them, construct 1–237 was selected for downstream applications because it encompasses key secondary structural features while avoiding the expression barriers caused by the highly disordered region at the C-terminus (Figure 4 and Figure S1C).

Subsequently, using this truncated antigen, hens were immunized to generate a VGLL3-specific avian antibody. SDS-PAGE of the purified avian antibody revealed distinct bands corresponding to the heavy and light chains (−66 and −23 kDa, respectively) (Figure 4C). Western blot analysis confirmed the specificity of the antibody, demonstrating concentration-dependent binding to VGLL3 (Figure 4D). These observations demonstrate that the purified avian antibody possesses high specificity and structural integrity, effectively recognizing the VGLL3 target antigen.

An ic-ELISA was developed and optimized with a coating antigen concentration of 10 µg/mL and an avian antibody dilution of 1:1600 (Figure S2). The assay exhibited high sensitivity and linearity (Figure 4E, n = 4), with a standard curve regression equation of Y = −0.304X + 1.0572 (R^2^ = 0.9956). The limit of detection and IC_50_ values were calculated as 12.55 nM and 257.05 nM, respectively.

To evaluate the antibody’s tissue specificity, immunohistochemical staining was performed on myocardial tissue sections from sham and myocardial infarction mouse models. In the sham group, myocardial fibers appeared well-organized with uniformly stained nuclei and negligible background, indicative of preserved tissue architecture (Figure 4F, upper panels I–III). In contrast, myocardial infarction hearts displayed typical fibrotic changes, including disrupted cardiomyocyte alignment, increased interstitial space, and nuclear irregularities. Robust VGLL3-positive staining as brown DAB signal was predominantly localized to fibrotic regions, underscoring the antibody’s selective binding to pathological tissues (Figure 4F, lower panels I–III). Quantitative analysis showed a significant increase in VGLL3-positive area in myocardial infarction hearts compared to controls (Figure 4G, *p* < 0.001). These results establish the VGLL3-specific avian antibody as a reliable, sensitive, and fibrosis-selective probe, offering a promising platform for early-stage fibrotic tissue detection.

Furthermore, to preliminarily assess the detectability of VGLL3 in body fluid samples, we attempted to detect VGLL3 protein expression in the plasma of patients with cirrhosis by Western blot. However, under current experimental conditions, we failed to obtain a clear positive signal (Figure S4B).

## 4. Discussion

**Figure 5:**
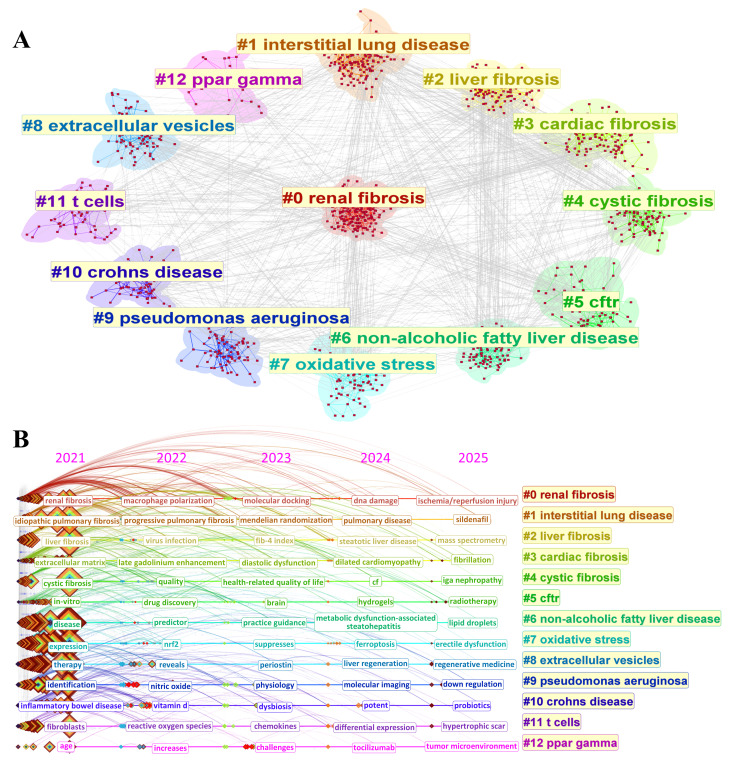
Keyword cluster analysis of literature related to fibrosis research (2021–2025). (**A**) Network visualization of keyword clustering, with different colors representing different fibrosis-related clustering results. (**B**) Temporal trends in fibrosis research, including annual data from 2021 to 2025.The data used were extracted from the Core Collection of Web of Science databases. The search strategy was as follows: TS = (fibrosis diseases OR fibrotic diseases) OR TI = (fibrosis diseases OR fibrotic diseases) OR AK = (fibrosis diseases OR fibrotic diseases) OR AB = (fibrosis diseases OR fibrotic diseases). The language type is limited to English, and the document type is limited to articles and reviews. The search spans from 2021 to 2025 (as of 27 March 2025).

Fibrosis is not a disease, but the result of tissue injury characterized by ECM deposition and irreversible organ remodeling. It is a pathological endpoint shared by diverse chronic diseases and is implicated in approximately 45% of deaths in industrialized nations [3]. As shown by keyword clustering analysis (Figure 5), recent fibrosis research has expanded beyond liver and lung involvement, encompassing renal fibrosis (#0), interstitial lung disease (#1), liver fibrosis (#2), cardiac fibrosis (#3), cystic fibrosis (#4), and exploring emerging mechanisms such as extracellular vesicle signaling, PPARγ modulation, oxidative stress, and immune regulation. These interdisciplinary themes reflect the systemic nature of fibrosis and underscore the need for integrated epidemiological, mechanistic, and diagnostic approaches.

Based on GBD 2021 data, our analysis presents a comprehensive global landscape of fibrosis-related DALYs and mortality across 204 countries from 1990 to 2021. During this period, the global burden of fibrotic diseases showed a steady upward trend, with DALYs increasing by 16.71% and mortality by 4.83% (Figure 1). Among the various fibrotic conditions, neoplasms and COPD emerged as the two most significant contributors to both DALYs and deaths. Despite their clinical differences, both diseases share progressive and irreversible fibrosis as a central pathological feature: COPD is characterized by scar formation in the small airways and lung interstitium, while neoplasms often involve paracancerous interstitial fibrosis [13,14]. This common fibrotic mechanism leads to structural damage, loss of organ function, and increased resistance to treatment, making neoplasms and COPD pivotal in fibrosis-related research. Furthermore, these two diseases are supported by robust global datasets on morbidity, mortality, and disability, ensuring the reliability of cross-national comparisons and trend analyses. From a public health standpoint, both disproportionately affect high-risk populations such as the elderly, smokers, individuals exposed to environmental hazards, and cancer survivors, thereby serving as crucial targets for policy-making, preventive strategies, and intervention evaluation.

Further stratified regional analysis showed that the disease burden of neoplasms and COPD showed significant geographical heterogeneity. Neoplasm-related DALYs remained disproportionately high in high-SDI countries, whereas COPD burden was concentrated in East and South Asia, sub-Saharan Africa, and low-SDI regions (Figure 2). While global neoplasm-related DALYs declined by 25.59%, reductions were most substantial in high-middle SDI countries, while low-middle and low SDI regions showed only modest improvements, highlighting persistent inequalities in healthcare access and treatment capacity. For COPD, although global ASR of DALYs fell by 36.98%, the burden remained more than three times higher in low-SDI countries compared to high-SDI countries in 2021, underscoring the disproportionate impact of environmental and behavioral risk factors.

Inequality metrics further underscore the divergence in global fibrosis dynamics. The slope index of inequality and concentration index of inequality for neoplasms decreased substantially, indicating narrowing absolute and relative disparities and a transition toward a more balanced global distribution (Figure 3A,B). In contrast, COPD showed worsening inequality: although slope index of inequality slightly improved, concentration index of inequality remains negative, indicating increasing concentration of COPD burden in low-SDI regions (Figure 3C,D). These findings underscore two contrasting trends: while equity in neoplasms-related disease burden has improved, disparities in COPD burden have continued to worsen and require urgent attention.

Furthermore, frontier analysis, comparing country-specific DALYs rates with the minimum achievable burden at a given SDI level, identified several under-resourced countries such as Bangladesh, Burkina Faso, and Guinea as outperforming expectations, closely aligning with the global optimal frontier (Figure 3F,H). These successes reflect the potential of scalable, low-cost interventions even under constrained conditions. Conversely, several high-SDI countries, such as the Netherlands, United States, and United Kingdom, continue to deviate substantially from the frontier, revealing inefficiencies despite resource abundance. These findings emphasize that closing the inequality gap will require both resource-sensitive innovation in low-SDI settings and systemic reform in high-SDI contexts.

Against this backdrop, a novel, sensitive, and context-appropriate diagnostic strategy is critically needed to complement current imaging and serum-based fibrosis assessments, which suffer from specificity limitations, technical dependence, or invasiveness [15,16]. Existing anti-fibrotic drugs such as nintedanib and pirfenidone can slow down the progression of fibrosis [17,18], but their mechanism of action relies on inhibiting pro-fibrotic signaling pathways (such as the TGF-β pathway) [19]. This may not only affect the normal tumor suppression function, but also cause adverse reactions such as gastrointestinal discomfort, bronchitis, and liver dysfunction [20,21]. In this study, we propose VGLL3 as a promising diagnostic and therapeutic target in fibrosis. VGLL3, a transcriptional co-regulator, has emerged as a pivotal factor in fibrotic processes. It regulates essential cellular functions, including proliferation, differentiation, apoptosis, and DNA repair, and is involved in fibrosis by responding to matrix stiffness and activating myofibroblasts [22,23,24]. Specifically, in the presence of matrix stiffness, VGLL3 translocates to the nucleus and incorporates in NONO condensates, binds to EWSR1, and represses *miR-29b*, promoting collagen expression [6]. Given these characteristics, VGLL3 presents a promising target for diagnostic and therapeutic fibrosis strategies.

Structural modeling using AlphaFold2 revealed that the VGLL3 protein contains numerous IDRs (Figure 4A). This structural feature may be the primary reason for the difficulty in efficiently expressing the full-length protein in *E. coli*. To overcome this problem, we designed multiple truncated constructs based on the structure prediction results. Through expression screening and immunoblot analysis, we identified the construct encompassing residues 1–237 (Trunc-3) as the optimal construct (Figure S1A,B). This construct exhibited good expressions in *E. coli*, retained the primary α-helical and β-sheet structural regions, and avoided the expression barrier caused by the highly disordered C-terminal region (Figure S1D).

To develop a sensitive detection platform, we produced VGLL3-specific avian antibodies in hens. Compared to mammalian IgG, avian antibodies offer unique advantages: lack of cross-reactivity with human Fc receptors, reduced background binding, and enhanced recognition of mammalian antigens due to phylogenetic divergence [16]. The resulting anti-VGLL3 avian antibody demonstrated high specificity, as confirmed by SDS-PAGE and Western blot (Figure 4C,D), and enabled the development of a highly sensitive ic-ELISA with a detection limit of 12.55 nM (Figure 4E). Although slightly less sensitive than established fibrosis biomarkers such as CTGF (11.4 nM) and collagen VI (2.76 nM) [25,26], VGLL3 has the unique advantage of serving as an early-stage fibrosis indicator due to its upstream regulatory role in collagen synthesis. However, VGLL3 is prone to degradation and aggregation during purification due to its IDRs nature, which may affect the sensitivity of antigen preparation and immunoassay performance [27].

Importantly, immunohistochemistry on myocardial infarction mouse models demonstrated that the anti-VGLL3 avian antibody selectively stains fibrotic tissues, with significantly stronger signals in myocardial infarction hearts compared to controls (*p* < 0.001; Figure 4F,G), confirms its in situ binding specificity and potential for tissue-level fibrosis detection. Given its low sequence similarity to other VGLL family proteins but high interspecies conservation (Figure S3), this antibody platform holds translational potential for broad cross-species applications.

In addition, the upstream regulatory role of VGLL3 in the development of fibrosis makes it a promising early diagnostic biomarker that can complement rather than replace existing markers. Multiplex detection strategies combining complement 3 (Pro-C3), connective tissue growth factor (CTGF) and type VI collagen can improve the temporal and spatial resolution of fibrosis progression. Compared with traditional single markers, VGLL3 can reflect earlier molecular changes, capturing abnormal protein expression prior to imaging findings, thereby improving sensitivity and reducing the risk of false negatives. Notably, VGLL3 is consistently expressed across multiple organs, potentially identifying systemic fibrosis [6]. Its tissue-level detection has good feasibility: tissue collection is standardized, and the technology is mature. Combined with the ic-ELISA platform we developed, the detection sensitivity is high, and the cost is low (about US$1–2 per sample), which is much lower than MRI and commercial serum kits, and can significantly reduce the detection cost (>60%) in resource-limited areas. This platform is suitable for grassroots laboratories, facilitating early screening of fibrosis in resource-limited areas, and helping to narrow the diagnostic gap.

Beyond fibrosis, VGLL3 is also widely expressed in various tissues and implicated in multiple disease processes, including cancer and autoimmune disorders. VGLL3 is overexpressed in cancer cells, such as those from breast tumors and osteosarcomas [22], and is involved in autoimmune diseases, including lupus, scleroderma, and Sjögren’s syndrome [28,29]. This shows its research value as a potential pan-biomarker. Our study marks a technical milestone, the first successful use of avian antibodies to target IDRs proteins. This expands the application range of avian antibody-based immunoassays to traditional structured targets [30,31]. This method not only provides key technical support for the subsequent quantitative analysis of VGLL3 in large-scale population samples but also expands the application potential of avian antibodies in the detection of high-risk proteins (such as TDP-43 and FUS) in neurodegenerative diseases and tumors [32,33,34].

While the VGLL3-based early fibrosis detection platform constructed in this study has achieved preliminary validation in a mouse model of myocardial infarction, it still has limitations. Tissue-level validation is currently limited to the heart and does not yet encompass common fibrotic organs such as the liver, lungs, and kidneys, limiting its cross-organ applicability. Western blot analysis did not detect VGLL3 protein signals in the plasma of patients with cirrhosis (Figure S4B), suggesting that its expression level in the blood is low or its stability is poor, which is not conducive to its application as a circulating biomarker. Furthermore, while the ic-ELISA platform offers advantages in cost and sensitivity, it remains in the laboratory stage and lacks systematic validation with clinical samples. Future studies should expand the validation of VGLL3 expression in multi-organ models, conduct in situ detection in human tissues, and evaluate its correlation with clinical markers. Furthermore, optimization of bodily fluid detection strategies (such as protein enrichment and mass spectrometry) should be performed to enhance detection sensitivity and stability.

In summary, our study integrates global epidemiological insights with biomolecular innovation to address the critical diagnostic challenges of fibrosis. We not only identify VGLL3 as a mechanistically meaningful and diagnostically relevant target but also establish a novel avian antibody-based immunoassay platform with broad translational potential. This work paves the way for future multidimensional fibrosis diagnostics and opens new avenues for the detection of disordered proteins in human disease.

## 5. Conclusions

This study provides an integrated global analysis of fibrosis-related disease burden using GBD 2021 data, revealing contrasting trends between neoplasms and COPD. While disparities in neoplasm-related DALYs have narrowed over time, the burden of COPD has become increasingly concentrated in low-SDI regions, underscoring persistent global health inequalities. Frontier efficiency analysis further identifies some low-SDI countries achieving near-optimal burden reduction despite limited resources. Importantly, we developed and validated a novel immunoassay based on avian antibodies, targeting the IDRs-containing protein VGLL3, a key upstream regulator of collagen synthesis. This represents the first successful application of avian antibodies for detecting IDRs proteins, offering enhanced specificity and early diagnostic potential for fibrosis. By integrating global epidemiological insights with an innovative diagnostic strategy, our work provides new tools and perspectives for advancing equitable and effective fibrosis detection and control.

## Figures and Tables

**Table 1:** Global and SDI regional age-standardized death and DALYs rates for neoplasms and COPD in 1990 and 2021. ASDR: age-standardized death rate.

	1990		2021	
Characteristics	ASDR per 100,000 (95% UI)	ASR of DALYs per 100,000 (95% UI)	ASDR per 100,000 (95% UI)	ASR of DALYs per 100,000 (95% UI)
neoplasms				
Global	148.24 (140.18, 154.39)	3969.21 (3792.05, 4135.06)	116.49 (107.28, 124.69)	2953.59 (2769.24, 3154.03)
High SDI	170.96 (162.88, 175.00)	4341.14 (4215.00, 4430.83)	123.22 (113.06, 128.96)	2920.60 (2751.48, 3031.34)
High-middle SDI	173.65 (163.76, 182.46)	4809.50 (4539.00, 5055.08)	134.01 (121.72, 147.09)	3388.27 (3078.37, 3728.81)
Middle SDI	135.62 (125.17, 146.36)	3778.75 (3482.10, 4077.32)	109.59 (99.35, 121.22)	2852.23 (2611.28, 3158.67)
Low-middle SDI	82.45 (75.29, 88.04)	2408.70 (2227.63, 2562.93)	85.03 (79.17, 90.89)	2376.66 (2202.92, 2542.90)
Low SDI	98.74 (87.84, 110.68)	2864.53 (2554.22, 3184.28)	90.64 (79.86, 102.00)	2487.39 (2164.52, 2827.72)
COPD				
Global	71.92 (64.47, 77.53)	1492.64 (1342.46, 1609.30)	45.22 (40.61, 49.70)	940.66 (871.48, 1014.59)
High SDI	25.53 (23.71, 26.50)	589.80 (557.84, 616.39)	19.44 (17.26, 20.66)	471.22 (437.45, 498.84)
High-middle SDI	79.53 (70.61, 86.61)	1511.32 (1365.74, 1635.67)	35.91 (30.78, 40.69)	691.14 (621.83, 772.74)
Middle SDI	123.89 (109.19, 134.80)	2332.91 (2063.49, 2546.31)	57.45 (49.59, 65.43)	1076.67 (963.62, 1201.24)
Low-middle SDI	92.07 (74.17, 107.46)	1963.19 (1602.24, 2252.75)	84.76 (75.80, 93.78)	1707.90 (1558.88, 1865.11)
Low SDI	77.67 (61.91, 91.44)	1673.81 (1373.37, 1936.99)	70.70 (63.35, 79.76)	1457.94 (1318.76, 1617.05)

## Data Availability

The data presented in this study are available in UniProt database at https://www.uniprot.org/ (accessed on 14 March 2024), reference number P85442.

## Supplementary Materials

The following supporting information can be downloaded at: https://www.mdpi.com/article/10.3390/biom15091273/s1, Figure S1. Expression and structural analysis of five VGLL3 truncation constructs. Figure S2. Antibody titer determination. Figure S3. VGLL3 protein sequence alignment. Figure S4. SDS-PAGE and western blot analysis of VGLL3 protein in collaborator’s blood samples. Table S1. Primer sequences for VGLL3 truncation constructs. Table S2. Regional and SDI-specific age-standardized death and DALYs rates for neoplasms in 1990 and 2021. Table S3. Regional and SDI-specific age-standardized death and DALYs rates for COPD in 1990 and 2021.

## Abbreviations

The following abbreviations are used in this manuscript:

GBD	global burden of disease
DALYs	disability-adjusted life years
ASR	age-standardized rate
SDI	socio-demographic index
VGLL3	vestigial-like family member 3
COPD	chronic obstructive pulmonary disease
ic-ELISA	indirect competitive enzyme-linked immunosorbent assay
ECM	extracellular matrix
IDRs	intrinsically disordered regions
