# Supplementary material for: Global Fibrosis Burden and a Transcriptional Biomarker-Based Strategy for Early Detection in Resource-Limited Settings
Source: Biomolecules. 2025 Sep 3;15(9):1273. doi: 10.3390/biom15091273 (PMC12467191; doi:10.3390/biom15091273)
Supplement: Supplementary file 1 [file biomolecules-15-01273-s001.zip › biomolecules-3767478-SI.pdf]

Supplementary

Global Fibrosis Burden and a Transcriptional Biomarker-Based Strategy for Early Detection in Resource-Limited Settings

Qinqin Deng [1], Longjiang Wu [1], Chenlu Zhang [1,2]* and Mei Dang [1]*

1 College of Biological Sciences and Engineering, Shaanxi University of Technology, Hanzhong 723000, China
2 Shaanxi Provincial Key Laboratory of Resource Biology, Shaanxi University of Technology, Hanzhong 723000, China

* Correspondence: chenluzhang@snut.edu.cn (C.L.Z.); e0269788@u.nus.edu (M.D.)

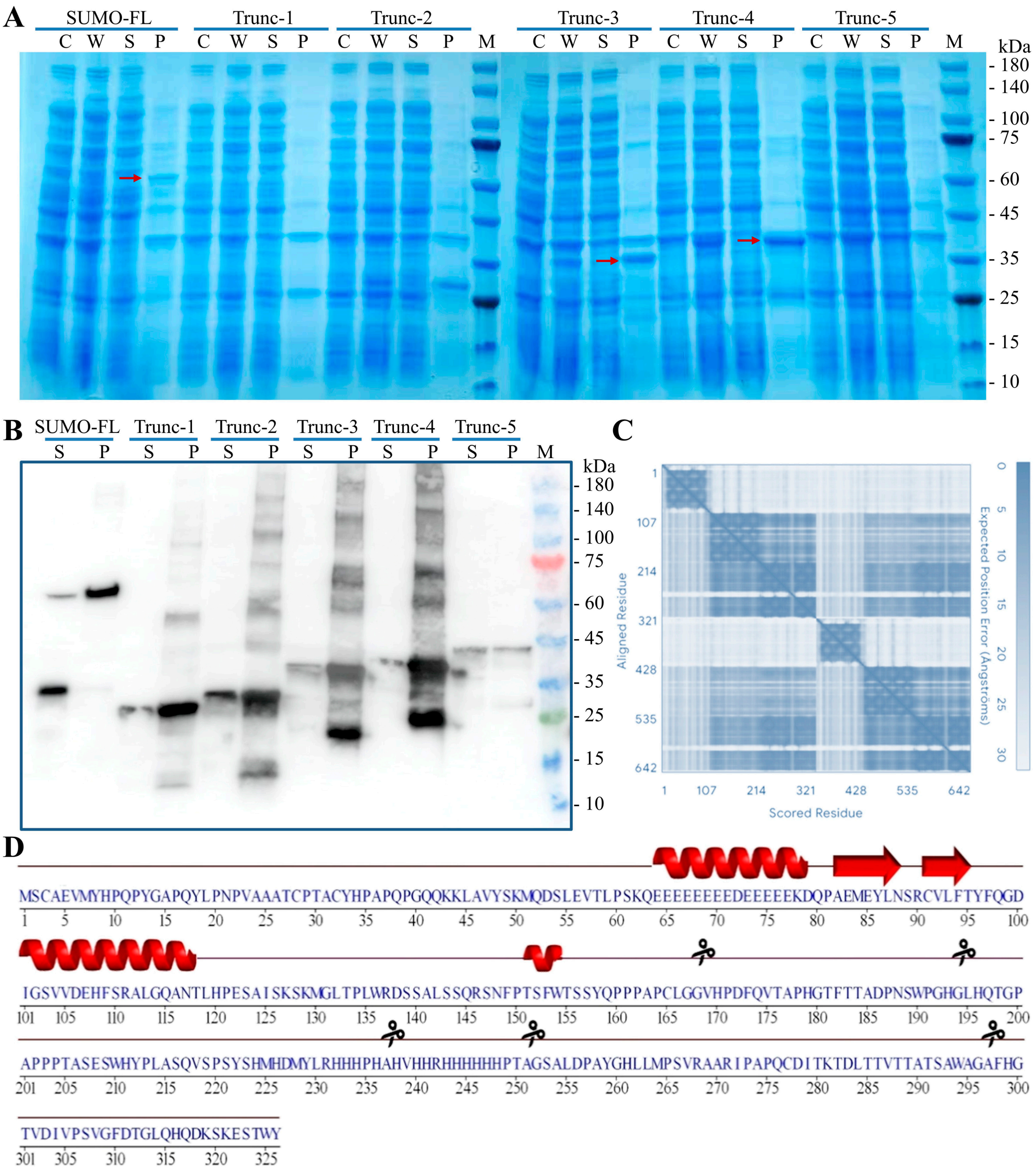

Figure S1. Expression and structural analysis of five VGLL3 truncation constructs

Notes: (A) SDS-PAGE expression analysis of SUMO-FL and five VGLL3 truncation constructs. M: protein marker. Lanes represent the uninduced control (C), whole cell lysate (W), soluble fraction (S), and precipitate (P), respectively. Red arrows indicate the target protein bands. (B) Expression of VGLL3 protein and its truncations was detected by western blot using an anti-His-tag antibody. M: protein marker. Lanes represent the soluble fraction (S) and precipitate (P), respectively. (C) Predicted alignment error map, generated by AlphaFold2, showing expected positional error between residue pairs in the protein sequence. (D) Amino acid sequence of VGLL3, with the α-helix and β-strand indicated by red helix and arrows, respectively. Truncation sites are marked with scissors.

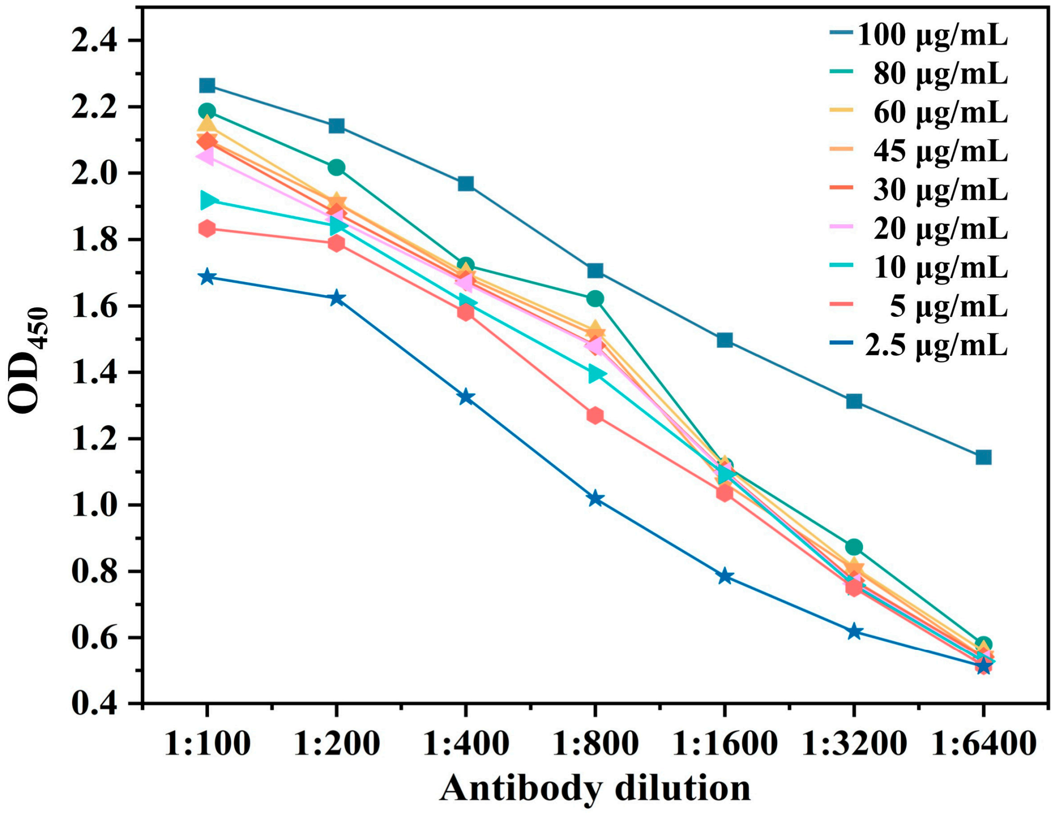

Figure S2. Antibody titer determination

Note: The line graph represents the reaction signal intensity (OD_{450} value) at different VGLL3 (residues 1-237) protein antigen concentrations (2.5-100 μg/mL) and anti-VGLL3 avian antibodies dilutions (1:100-1:6400).

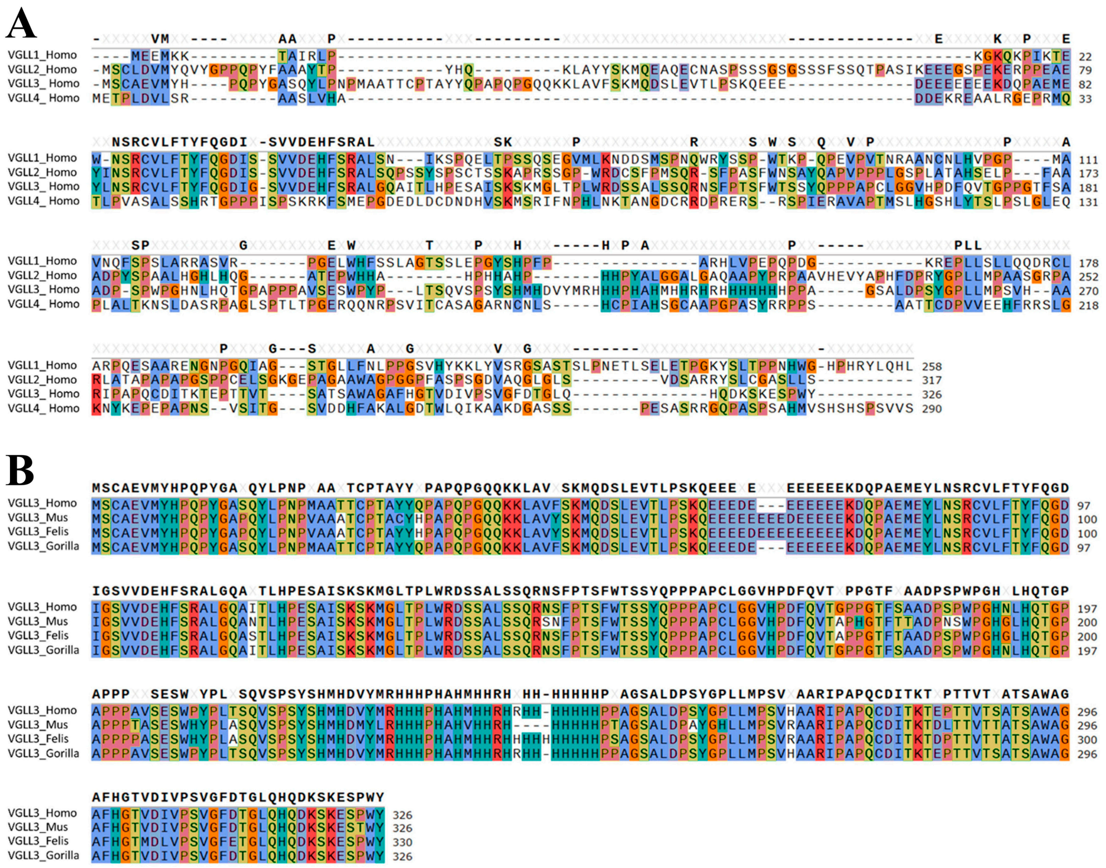

Figure S3. VGLL3 protein sequence alignment

Notes: (A) Sequence alignment of human VGLL3 with other members. (B) Sequence alignment of VGLL3 homologs from other species.

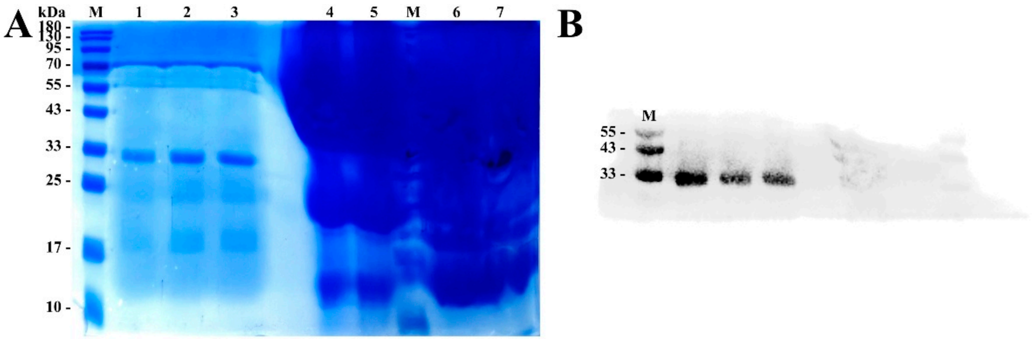

Figure S4. SDS-PAGE and western blot analysis of VGLL3 protein in collaborator's blood samples. (A) Lane M: protein marker. Lanes 1-3: SDS-PAGE analysis of purified VGLL3 (residues 1-237), with protein loading of 10, 15, and 25 µL, respectively. Lanes 4-5, 6-7 are SDS-PAGE analysis of plasma samples from two patients with cirrhosis, with loading of 25 and 35 µL, respectively. (B) Western blot analysis of anti-VGLL3 avian antibodies specificity.

Table S1. Primer sequences for VGLL3 truncation constructs

Serial number	Primer direction	primer sequences (5'-3')
Trunc-1	R	CCGCTCGAGACCGCCCAGGCACGGC
Trunc-2	R	CCGCTCGAGGCCATGACCAGGCCATGAGTTCG
Trunc-3	R	CCGCTCGAGTGCGTGCGGGTGGTGGTGG
Trunc-4	R	CCGCTCGAGCGCCGTCGGGTGGTGGTG
Trunc-5	R	CCGCTCGAGCGCGCCTGCCCAGGCG
Trunc-1-5	F	CATGCCATGGGTATGAGCTGCGCGGAAGTGATGTACC

Notes: Lists the serial numbers, primer directions (R for reverse, F for forward), and corresponding primer sequences (5'-3'). The restriction sites are underlined.

Table S2. Regional and SDI-specific age-standardized death and DALYs rates for neoplasms in 1990 and 2021

Characteristics	1990		2021	
	ASDR per100000 (95% UI)	ASR of DALYs per 100000 (95% UI)	ASDR per100000 (95% UI)	ASR of DALYs per 100000 (95% UI)
**Central Europe, eastern Europe, and central Asia**				
Central Asia	141.60(135.87, 147.20)	4191.12(4043.84, 4343.53)	93.71(85.59, 101.82)	2641.36(2400.47, 2873.43)
Central Europe	174.86(169.90, 178.57)	4759.08(4661.63, 4851.19)	157.77(146.33, 167.79)	3945.02(3668.14, 4199.24)
Eastern Europe	159.70(155.78, 162.62)	4694.70(4598.26, 4778.87)	126.36(116.53, 136.03)	3442.79(3180.11, 3726.83)
**High income region**				
High-income Asia Pacific	154.84(146.34, 159.82)	3895.49(3748.51, 3995.76)	110.36(97.95, 117.01)	2496.98(2312.21,2607.65)
High-income North America	170.66(161.94, 175.39)	4419.14(4270.09, 4524.11)	120.58(111.02, 125.99)	2941.27(2790.57,3052.17)
Western Europe	175.44(167.49, 179.58)	4395.62(4267.99, 4479.59)	125.84(115.27, 131.43)	2979.74(2805.61,3083.32)
Australasia	168.60(160.17, 173.83)	4214.85(4069.98, 4336.53)	117.46(107.55, 123.90)	2776.10(2607.21,2898.02)
**Latin America and Caribbean**				
Andean Latin America	130.67(120.12, 141.99)	3459.56(3191.42, 3756.09)	113.15(93.66, 135.67)	2884.25(2373.08, 3491.55)
Caribbean	135.55(128.97, 140.68)	3540.54(3346.04, 3712.70)	126.01(112.21, 140.08)	3293.53(2908.25, 3718.32)
Southern Latin America	172.70(165.73, 177.78)	4441.25(4314.00, 4559.02)	129.21(120.74, 135.27)	3212.04(3060.24, 3343.29)
Tropical Latin America	125.06(118.81, 128.35)	3332.12(3225.85, 3402.93)	111.43(103.88, 116.21)	2932.48(2796.48, 3032.81)
Central Latin America	115.00(110.90, 117.15)	3017.95(2948.21, 3064.98)	94.11(84.15, 104.83)	2533.32(2262.84, 2838.36)
**North Africa and Middle East**				
North Africa and Middle East	97.14(89.25, 105.51)	2670.35(2463.00, 2885.94)	90.11(80.96, 99.52)	2321.55(2069.48, 2571.30)
**South Asia**				
South Asia	74.80(67.51, 80.92)	2247.46(2040.71, 2425.36)	74.73(68.77, 81.23)	2128.19(1956.29, 2307.38)
**Southeast Asia, east Asia, and Oceania**				
East Asia	183.38(162.07, 205.67)	5095.37(4492.56, 5719.36)	137.08(115.32, 162.11)	3466.88(2919.94, 4109.42)
Oceania	98.06(78.65, 121.21)	2649.49(2081.98, 3304.41)	93.04(75.73, 113.59)	2491.25(2008.81, 3054.86)
Southeast Asia	99.18(90.20, 107.16)	2821.94(2549.35, 3069.41)	100.50(88.27, 111.20)	2725.67(2411.10, 3024.15)
**Sub-Saharan Africa**				
Central Sub-Saharan Africa	107.59(89.91, 129.75)	2919.23(2442.78, 3442.36)	100.35(80.35, 126.63)	2650.01(2104.34, 3326.27)
Eastern Sub-Saharan Africa	127.30(114.34, 139.87)	3688.66(3335.89, 4044.95)	113.98(100.35, 130.27)	3114.48(2700.69, 3622.80)
Southern Sub-Saharan Africa	118.39(107.48, 130.72)	3195.67(2936.22, 3513.52)	144.18(132.84, 154.13)	3804.26(3484.46, 4116.06)
Western Sub-Saharan Africa	81.44(67.58, 93.94)	2205.40(1839.49, 2549.58)	89.08(72.14, 103.40)	2269.98(1769.61, 2701.95)

Table S3. Regional and SDI-specific age-standardized death and DALYs rates for COPD in 1990 and 2021

Characteristics	1990		2021	
	ASDR per100000 (95% UI)	ASR of DALYs per 100000 (95% UI)	ASDR per100000 (95% UI)	ASR of DALYs per 100000 (95% UI)
Central Europe, eastern Europe, and central Asia				
Central Asia	36.33(34.02, 38.45)	811.63(767.26, 853.69)	21.27(19.12, 23.46)	498.60(452.01, 546.10)
Central Europe	30.65(29.40, 31.73)	679.30(652.23, 705.63)	16.03(14.70, 17.22)	413.12(382.55, 444.56)
Eastern Europe	35.77(34.15, 36.74)	788.92(757.69, 815.47)	11.72(10.79, 12.62)	324.42(301.04, 350.10)
High income region				
High-income Asia Pacific	14.17(12.76, 15.08)	311.78(286.99, 335.85)	6.68(5.70, 7.37)	182.14(162.64, 202.95)
High-income North America	26.28(24.30, 27.29)	703.28(663.83, 742.00)	29.89(26.20, 31.63)	736.05(685.79, 776.49)
Western Europe	24.66(22.93, 25.60)	520.26(495.21, 542.90)	17.82(15.74, 18.92)	390.81(361.46, 415.88)
Australasia	29.42(27.56, 30.92)	610.45(577.57, 639.32)	18.89(16.66, 20.42)	376.20(343.09, 400.39)
Latin America and Caribbean				
Andean Latin America	19.44(16.88, 21.80)	375.29(331.38, 416.67)	13.40(10.93, 16.29)	261.42(222.30, 306.62)
Caribbean	18.48(16.10, 20.50)	396.98(342.42, 442.74)	19.85(17.47, 22.49)	439.53(388.88, 499.30)
Southern Latin America	26.92(25.28, 28.40)	554.35(527.28, 583.08)	22.53(20.53, 24.07)	436.03(405.72, 461.73)
Tropical Latin America	44.64(41.13, 46.88)	880.79(834.58, 919.82)	26.32(23.44, 27.99)	554.73(513.83, 585.11)
Central Latin America	35.73(33.58, 36.90)	660.88(632.68, 684.66)	26.92(23.70, 29.64)	512.35(462.65, 560.08)
North Africa and Middle East				
North Africa and Middle East	36.17(28.39, 41.23)	794.61(655.01, 882.01)	26.37(23.19, 29.31)	599.18(546.07, 658.22)
South Asia				
South Asia	114.54(89.16, 136.08)	2424.19(1918.36, 2829.14)	101.63(90.55, 114.34)	2049.22(1862.71, 2268.73)
Southeast Asia, east Asia, and Oceania				
East Asia	225.27(194.01, 250.33)	3760.90(3272.11, 4173.78)	72.20(59.32, 85.26	1217.69(1043.87, 1422.79)
Oceania	144.27(113.03, 180.83)	2919.73(2293.23, 3660.43)	118.21(96.32, 144.59)	2351.49(1931.26, 2854.06)
Southeast Asia	60.18(48.55, 68.27)	1255.60(1042.88, 1403.69)	43.14(38.24, 48.54)	914.73(822.27, 1016.35)
Sub-Saharan Africa				
Central Sub-Saharan Africa	52.33(36.88, 72.56)	1150.66(851.36, 1503.88)	42.82(29.63, 65.02)	975.91(720.50, 1344.93)
Eastern Sub-Saharan Africa	42.29(32.10, 48.71)	959.70(762.97, 1088.96)	29.91(24.41, 35.14)	713.24(593.00, 816.07)
Southern Sub-Saharan Africa	37.40(32.85, 44.78)	896.69(806.97, 1006.53)	34.78(31.93, 37.34)	864.13(800.37, 929.92)
Western Sub-Saharan Africa	28.53(22.44, 33.71)	681.87(558.27, 780.37)	22.07(19.45, 25.05)	567.27(510.78, 631.22)

Article original image

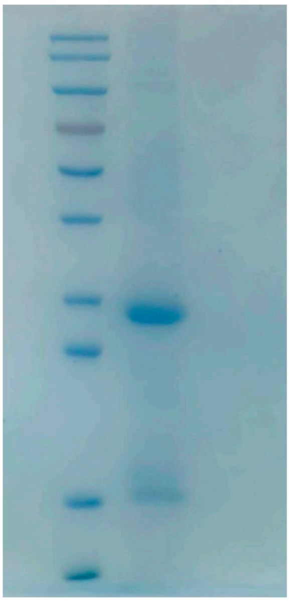

Note: SDS-PAGE analysis of the original purified VGLL3 (residues 1-237). Located in the manuscript Figure 4B.

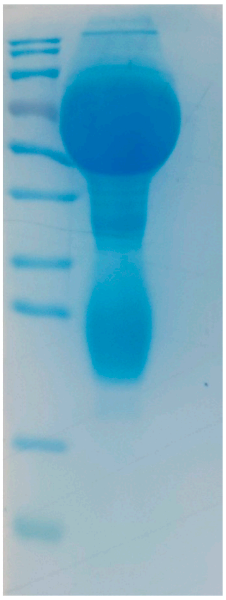

Note: Original SDS-PAGE analysis of purified anti-VGLL3 IgY antibody. Located in the manuscript Figure 4C.

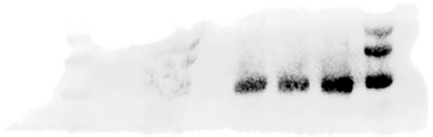

Note: Original image of Western blot analysis of anti-VGLL3 IgY specificity. Located in the manuscript Figure 4D.

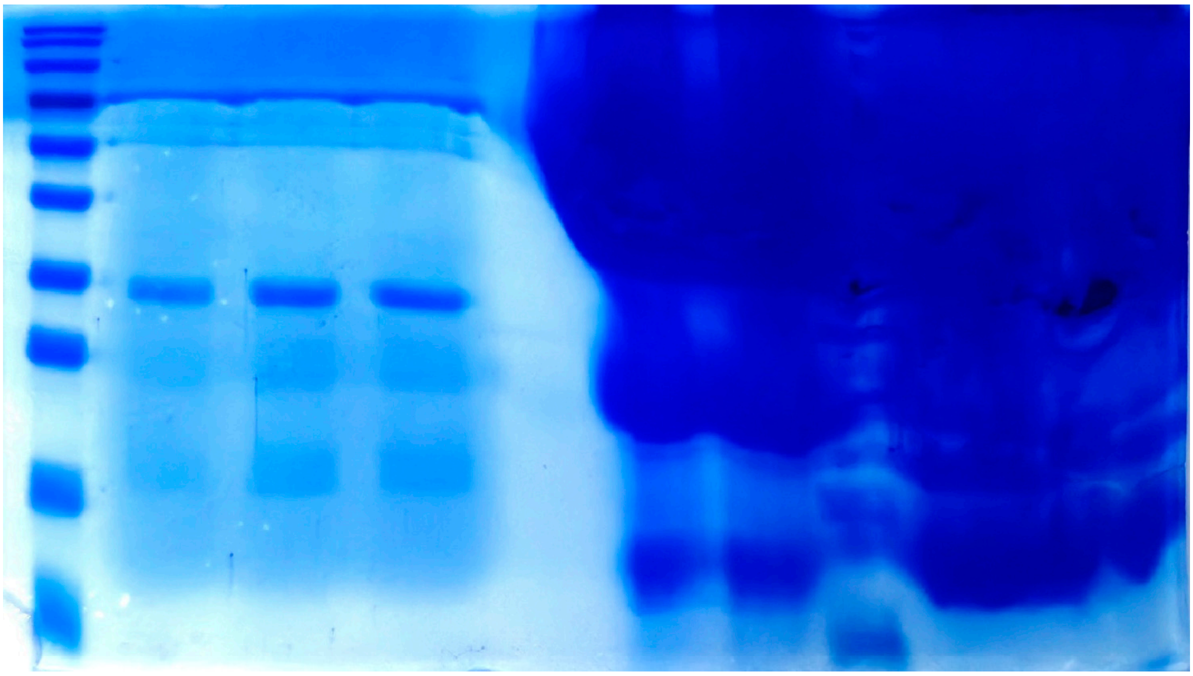

Note: Original image of SDS-PAGE analysis of VGLL3 protein in collaborator's blood sample. Located in the manuscript Figure S4A.
